# Association between low back pain and functional disability in the elderly people: a 4-year longitudinal study after the great East Japan earthquake

**DOI:** 10.1186/s12877-022-03655-7

**Published:** 2022-12-02

**Authors:** Yutaka Yabe, Yoshihiro Hagiwara, Yumi Sugawara, Ichiro Tsuji

**Affiliations:** 1 Department of Orthopedic Surgery, Tohoku University School of Medicine, 1-1 Seiryo-machi, Aoba-ku, Sendai, Miyagi 980-8574 Japan; 2 Department of Health Informatics and Public Health, Division of Epidemiology, Tohoku University Graduate School of Public Health, 2-1 Seiryo-machi, Aoba-ku, Sendai, Miyagi 980-8575 Japan

**Keywords:** Low back pain, Low physical function, Natural disaster, Great East Japan earthquake, Longitudinal study, Cohort duration frequency

## Abstract

**Background:**

Functional disability is a major health issue in an aging population. Low back pain (LBP) is a common health concern that can lead to functional disability in the elderly; nonetheless, their association has not yet been clarified. This study aimed to examine the association between LBP and functional disability in the elderly, with a focus on its dose-dependent effects.

**Methods:**

This study used the 4-year longitudinal data of people living in disaster-affected areas after the Great East Japan Earthquake (aged ≧65, *n* = 914). LBP and physical function were assessed at 2, 4, and 6 years after the disaster. Multivariate logistic regression analyses were performed to assess the association between LBP and low physical function, as well as the effect of preceding LBP on the onset of low physical function.

**Results:**

LBP was significantly associated with low physical function, and the association became stronger as the duration of LBP increased. Adjusted odds ratios (95% confidence intervals) were 1.27 (0.79–2.06) in “< 2 years,” 1.95 (1.01–3.77) in “≥2 years and <4 years,” and 2.34 (1.35–4.06) in “≥4 years” (*p* for trend = 0.009). Additionally, preceding LBP was significantly associated with the onset of low physical function, and the effect became prominent as the duration of LBP increased. Adjusted odds ratios (95% confidence intervals) were 2.28 (1.19–4.37) in “< 2 years” and 2.82 (1.35–5.90) in “≥2 years” (*p* for trend = 0.003).

**Conclusions:**

LBP is associated with physical disability among the elderly in a dose-dependent manner. Therefore, prevention and treatment of LBP are important for preventing functional disability.

**Supplementary Information:**

The online version contains supplementary material available at 10.1186/s12877-022-03655-7.

## Background

With an increase in the aging population, functional disability is currently a major concern globally [1–3]. Functional disability leads to a decline in activities of daily living and increases the need for long-term nursing care for the elderly. Hence, understanding the factors associated with functional disability is important for their prevention and for successful aging [3–5]. Several factors such as age, medical history, body mass index (BMI), frequency of going outdoors, and depression are reported to be associated with functional disability [6]. Further, musculoskeletal pain is a common symptom in the elderly and is considered to be a major cause of functional disability [7]. Due to its high prevalence and persistence, low back pain (LBP) is particularly regarded as the leading cause of functional disability in the elderly [8]. The effects of LBP on disability in the working population have been well described [9, 10]; on the other hand, those in the elderly have been gaining attention in recent times, and these reports have been gradually accumulating [2, 11–14]. Clarifying the association between LBP and functional disability is important for the development of strategies for preventing and resolving these problems. In particular, rehabilitation is beneficial to improve the elderly’s functional ability [15] and is also useful in pain treatment [16, 17]. However, a long-term longitudinal study concerning LBP and functional disability is rare, and the association between LBP and functional disability has not yet been clarified.

Functional disability in the elderly is also a major consequence of natural disasters [18–20]. Factors such as psychological distress and living conditions have been reported to be associated with the onset of functional disability after natural disasters [21, 22]. Further, natural disasters change living conditions and deprive the elderly of connections with local communities, leading to less frequency of going outdoors and a decrease in physical activities, thereby resulting in functional disability [23–25]. Reports on musculoskeletal pain and functional disability after natural disasters are rare; however, preceding musculoskeletal pain has been shown by a previous 1-year longitudinal study to be associated with the onset of functional disability [26]. Among people living in disaster-affected areas, LBP is the most common musculoskeletal pain, and its long-term effect on functional disability is a major concern [27, 28]. Therefore, the present study aimed to clarify the association between LBP and physical function using the 4-year longitudinal data of the elderly living in disaster-affected areas, with a particular focus on the dose-dependent association between LBP and physical function.

## Methods

### Participants

**Fig. 1:**
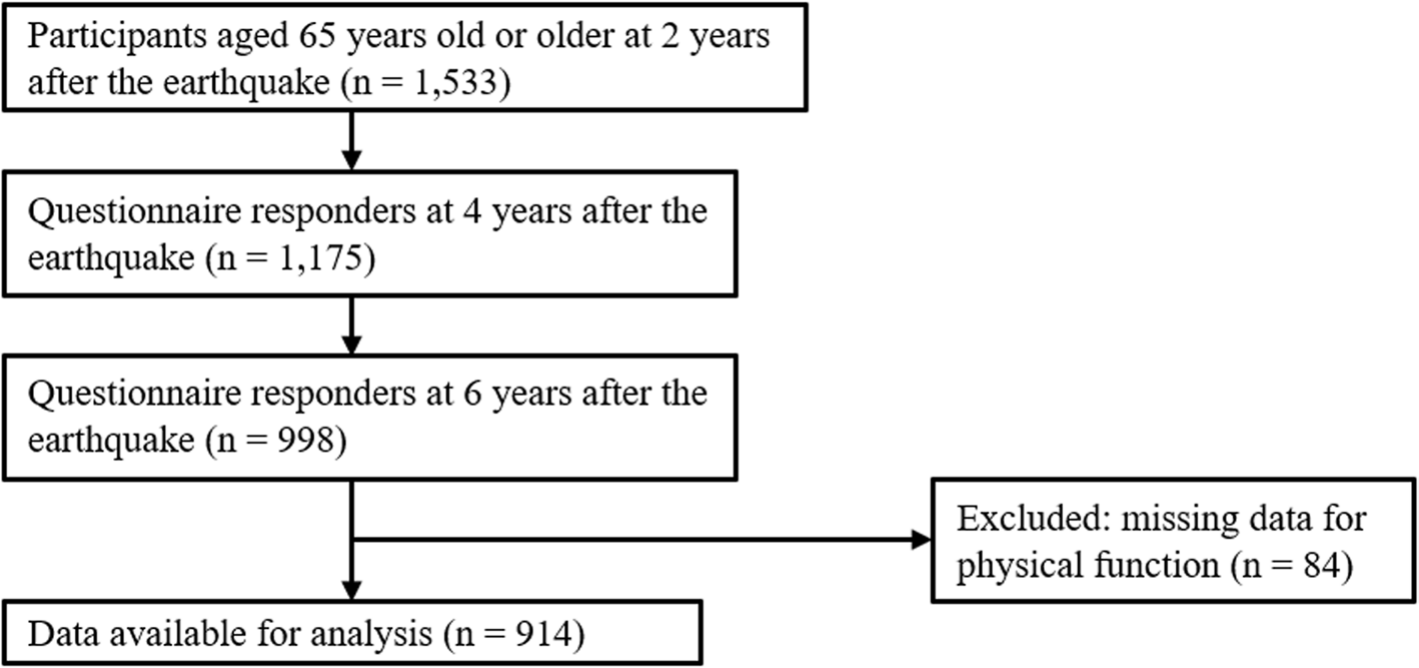
Flow chart of the study

This study used the longitudinal data of people living in disaster-affected areas after the Great East Japan Earthquake (GEJE). The GEJE was a catastrophic event in 2011 that caused severe damage to the northeastern coastal regions of Japan [29]. A panel survey was conducted on people living in these areas, including the Ogatsu, Oshika, and Ajishima areas in Ishinomaki City and Wakabayashi ward in Sendai City, Miyagi Prefecture [28]. The survey was first conducted at 3 months after the disaster and then repeated every year. The study population included all residents of the Ogatsu, Oshika, and Ajishima areas and people living in prefabricated housing in Wakabayashi ward. The present study used the data of participants aged ≥65 years at 2, 4, and 6 years after the disaster. At 2 years after the disaster (first time point), 1533 participants participated in this survey. Of these participants, 1175 (76.6%) responded to the survey held at 4 years after the disaster (second time point), and 998 (84.9%) responded at 6 years after the disaster (third time point). Participants with missing data on physical function were excluded. Finally, a total of 914 participants were enrolled in the present study (Fig. 1).

### LBP

LBP was assessed using a self-report questionnaire (Supplementary Material). The participants were asked whether they had experienced some symptoms in the last few days and were instructed to check the symptoms that they experienced; multiple symptoms could be chosen. The 28 choices included palpitation, dizziness, anorexia, hand or foot pain, knee pain, and LBP. The participants who checked LBP were considered to have LBP at each time point [30]. The duration of LBP at the third time point was defined and categorized into four groups: “absence,” absence of LBP at the third time point; “< 2 years,” absence of LBP at the second time point and presence of LBP at the third time point; “≥2 years and <4 years,” absence of LBP at the first time point and presence of LBP at the second and third time points; and “≥4 years,” presence of LBP at the first, second, and third time points. The frequency of LBP at the third time point was defined as the number in the presence of LBP at the first, second, and third time points and was categorized into four groups (absence, 1, 2, and 3). Further, the duration of LBP at the second time point was defined and categorized into three groups: “absence,” absence of LBP at the second time point; “< 2 years,” absence of LBP at the first time point and presence of LBP at the second time point; and “≥2 years,” presence of LBP at the first and second time points. The frequency of LBP at the second time point was defined as the number in the presence of LBP at the first and second time points and was categorized into three groups (absence, 1, and 2).

### Physical function

Physical function was assessed using the physical function score of the Kihon Checklist (KCL). The KCL is a self-report questionnaire used to identify frail people at risk of long-term nursing care [4]. The physical function score of the KCL comprises the following five yes/no questions, with each negative response receiving one point: (1) “Can you climb stairs without holding onto a handrail or wall?”; (2) “Can you get up from a chair without grabbing something?”; (3) “Are you able to walk for about 15 min?”; (4) “Have you fallen in the past year?”; and (5) “Are you very worried about falling?”. Low physical function was defined as a score of ≥3/5 in the physical function score of the KCL [4, 23].

### Covariates

The following variables assessed at the second or third time point were used in the analysis as covariates because they could be confounding factors: sex, age, BMI, living area, smoking and drinking habits, comorbidities, employment status, walking duration/day, living status, economic condition, psychological condition, sleep condition, and social network. Psychological condition, sleep condition, and social network were assessed using the Kessler Psychological Distress Scale [31], Athens Insomnia Scale [32], and Lubben Social Network Scale [33]. Psychological distress, sleep disturbance, and social isolation were defined as scores of ≥10/24, ≥6/24, and < 12/30, respectively. The variables were categorized, as shown in Table 1.

**Table 1:** Baseline characteristics

	Low back pain			
	n (%)	absence	presence	*P* value
	914	653	261	
Sex				
Male	396 (43.3)	289 (44.3)	107 (41.0)	0.369
Female	518 (56.7)	364 (55.7)	154 (59.0)	
Age				
< 75	276 (30.2)	200 (30.6)	76 (29.1)	0.654
≥ 75	638 (69.8)	453 (69.4)	185 (70.9)	
Body mass index^a^				
≥ 18.5, < 25	530 (58.0)	384 (58.8)	146 (55.9)	0.048
< 18.5	27 (3.0)	24 (3.7)	3 (1.1)	
≥ 25	308 (33.7)	207 (31.7)	101 (38.7)	
Living area				
Ogatsu	396 (43.3)	279 (42.7)	117 (44.8)	0.508
Oshika	301 (32.9)	221 (33.8)	80 (30.7)	
Ajishima	88 (9.6)	66 (10.1)	22 (8.4)	
Wakabayashi	129 (14.1)	87 (13.3)	42 (16.1)	
Smoking habits^a^				
Non-smoker	799 (87.4)	581 (89.0)	218 (83.5)	0.049
Smoker	60 (6.6)	40 (6.1)	20 (7.7)	
Drinking habits^a^				
Non-drinker	581 (63.6)	408 (62.5)	173 (66.3)	0.088
< 45.6 g of alcohol/day^b^	164 (17.9)	130 (19.9)	34 (13.0)	
≥ 45.6 g of alcohol/day^b^	46 (5.0)	30 (4.6)	16 (6.1)	
Comorbid conditions				
Hypertension	539 (59.0)	366 (56.0)	173 (66.3)	0.004
Diabetes mellitus	119 (13.0)	81 (12.4)	38 (14.6)	0.382
Myocardial infarction	104 (11.4)	61 (9.3)	43 (16.5)	0.002
Cerebral stroke	28 (3.1)	18 (2.8)	10 (3.8)	0.394
Working status^a^				
Unemployed	696 (76.1)	503 (77.0)	193 (73.9)	0.103
Employed	179 (19.6)	128 (19.6)	51 (19.5)	
Walking time/day^a^				
≥ 1 h	201 (22.0)	154 (23.6)	47 (18.0)	0.013
30 min to < 1 h	353 (38.6)	257 (39.4)	96 (36.8)	
< 30 m	333 (36.4)	219 (33.5)	114 (43.7)	
Living status^a^				
Same house as before the GEJE	299 (32.7)	222 (34.0)	77 (29.5)	0.375
Prefabricated house	83 (9.1)	54 (8.3)	29 (11.1)	
New house	242 (26.5)	177 (27.1)	65 (24.9)	
Others	254 (27.8)	174 (26.6)	80 (30.7)	
Economic condition^a^				
Normal	503 (55.0)	374 (57.3)	129 (49.4)	0.003
A little hard	195 (21.3)	140 (21.4)	55 (21.1)	
Hard	142 (15.5)	86 (13.2)	56 (21.5)	
Very hard	52 (5.7)	33 (5.1)	19 (7.3)	
Psychological distress^a^				
Absence	774 (84.7)	573 (87.7)	201 (77.0)	< 0.001
Presence	108 (11.8)	55 (8.4)	53 (20.3)	
Sleep disturbance^a^				
Absence	605 (66.2)	483 (74.0)	122 (46.7)	< 0.001
Presence	303 (33.2)	164 (25.1)	139 (53.3)	
Social isolation^a^				
Absence	702 (76.8)	507 (77.6)	195 (74.7)	0.31
Presence	209 (22.9)	143 (21.9)	66 (25.3)	

^a^Because each item has a limited number of respondents, the actual number is not necessarily in accordance with the total

^b^22.8 g of alcohol amount to 1 go or traditional unit of sake (180 ml), which also approximates to two glasses of wine (200 ml), or beer (500 ml) in terms of alcohol content. Categorical values are presented as numbers and percentage (%)

*GEJE* Great East Japan Earthquake

### Statistical analysis

The χ^2^ test was performed to compare the covariates due to the presence of LBP at the third time point. Crude and multivariate logistic regression analyses were performed to assess the association between LBP and low physical function at the third time point, and odds ratios (ORs) and 95% confidence intervals (95% CIs) were calculated. Further, the association between the duration or frequency of LBP and low physical function at the third time point was assessed using the same method. Covariates were set as variables at the third time point.

To assess the association between preceding LBP and the onset of low physical function, participants without low physical function at the second time point were selected (*n* = 615). The association between LBP at the second time point and low physical function at the third time point was also assessed. The association between the duration or frequency of LBP at the second time point and low physical function at the third time point was assessed using the same method. The covariates were set as variables at the second time point. All statistical analyses were performed using SPSS version 24.0 (IBM Corp., Armonk, NY, USA), with statistical significance set at *p* <  0.05.

## Results

The baseline characteristics of the participants at the third time point are presented in Table 1. Among the 914 participants, 261 (28.6%) had LBP. Participants with LBP were likely to have a high BMI, smoking habits, comorbidities, such as hypertension and myocardial infarction, shorter walking time, poor economic condition, psychological distress, and sleep disturbance. The proportion of participants with low physical function was 35.7% (326/914). LBP was significantly associated with low physical function, with an adjusted OR (95% CI) of 1.70 (1.18–2.44). Further, the duration of LBP was significantly associated with low physical function and the adjusted ORs (95% CIs) were 1.27 (0.79–2.06) in “< 2 years,” 1.95 (1.01–3.77) in “≥2 years and <4 years,” and 2.34 (1.35–4.06) in “≥4 years” when “absence” was used as a reference (*p* for trend = 0.009; Table 2). Moreover, the frequency of LBP was significantly associated with low physical function and the adjusted ORs (95% CIs) were 1.77 (1.17–2.69) in “1,” 1.74 (1.09–2.77) in “2,” and 2.79 (1.58–4.93) in “3” when “absence” was used as a reference (*p* for trend = 0.001; Table 3).

**Table 2:** Association between low back pain and physical function

		Low back pain				
	Total	Absence	Presence			*P* value
Participants	914	653	261			
Low physical function, n (%)	326 (35.7)	199 (30.5)	127 (48.7)			
Adjusted OR (95%CI)		1 (Ref.)	1.70 (1.18–2.44)			0.004
			Duration			
			< 2 years	^3^2 years, <4 years	^3^4 years	P for trend
Participants			118	54	89	
Low physical function, n (%)			47 (39.8)	30 (43.8)	50 (56.2)	
Adjusted OR (95%CI)		1 (Ref.)	1.27 (0.79–2.06)	1.95 (1.01–3.77)	2.34 (1.35–4.06)	0.009

Adjusted for sex, age, body mass index, living area, smoking habits, drinking habits, comorbid conditions, working status, walking time, living status, subjective economic condition, psychological distress, sleep disturbance, and social isolation. *OR* Odds ratio, *CI* Confidence interval

**Table 3:** Association between the frequency of low back pain and physical function

	Frequency of low back pain					
	Total	Absence	1	2	3	P for trend
Participants	914	496	181	148	89	
Low physical function, n (%)	326 (35.7)	134 (27.0)	73 (40.3)	69 (46.6)	50 (56.2)	
Crude OR (95%CI)		1 (Ref.)	1.83 (1.28–2.61)	2.36 (1.62–3.45)	3.46 (2.18–5.50)	< 0.001
Adjusted OR (95%CI)		1 (Ref.)	1.77 (1.17–2.69)	1.74 (1.09–2.77)	2.79 (1.58–4.93)	0.001

Adjusted for sex, age, body mass index, living area, smoking habits, drinking habits, comorbid conditions, working status, walking time, living status, subjective economic condition, psychological distress, sleep disturbance, and social isolation. *OR* Odds ratio, *CI* Confidence interval

Among participants without low physical function at the second time point, the new onset of low physical function at the third time point was 18.2% (112/615). Preceding LBP was significantly associated with new onset of low physical function, and the adjusted OR (95% CI) was 2.50 (1.47–4.23). Further, the duration of LBP at the second time point was significantly associated with the onset of low physical function and adjusted ORs (95% CIs) were 2.28 (1.19–4.37) in “< 2 years” and 2.82 (1.35–5.90) in “≥2 years” when “absence” was used as a reference (*p* for trend = 0.003; Table 4). Moreover, the frequency of preceding LBP was significantly associated with the new onset of low physical function and the adjusted ORs (95% CIs) were 2.44 (1.42–4.19) in “1” and 3.33 (1.56–7.10) in “2” when “absence” was used as a reference (*p* for trend = 0.001; Table 5).

**Table 4:** Association between preceding low back pain and onset of low physical function

	Low back pain at the second time point				
	Total	Absence	Presence		*P* value
Participants without low physical function at the second time point	615	484	131		
Onset of low physical function at the third time point, n (%)	112 (18.2)	69 (14.3)	43 (32.8)		
Adjusted OR (95%CI)		1 (Ref.)	2.50 (1.47–4.23)		0.001
			Duration		
			< 2 years	^3^2 years	P for trend
Participants without low physical function at the second time point			69	62	
Onset of low physical function at the third time point, n (%)			23 (33.3)	20 (32.3)	
Adjusted OR (95%CI)		1 (Ref.)	2.28 (1.19–4.37)	2.82 (1.35–5.90)	0.003

Adjusted for sex, age, body mass index, living area, smoking habits, drinking habits, comorbid conditions, working status, walking time, living status, subjective economic condition, psychological distress, sleep disturbance, and social isolation. *OR* Odds ratio, *CI* Confidence interval

**Table 5:** Association between the frequency of preceding low back pain and onset of low physical function

	Frequency of low back pain at the second time point				
	Total	Absence	1	2	P for trend
Participants without low physical function at the second time point	615	418	135	62	
Onset of low physical function at the third time point, n (%)	112 (18.2)	53 (12.7)	39 (28.9)	20 (32.3)	
Crude OR (95%CI)		1 (Ref.)	2.80 (1.75–4.48)	3.28 (1.79–6.01)	< 0.001
Adjusted OR (95%CI)		1 (Ref.)	2.44 (1.42–4.19)	3.33 (1.56–7.10)	0.001

Adjusted for sex, age, body mass index, living area, smoking habits, drinking habits, comorbid conditions, working status, walking time, living status, subjective economic condition, psychological distress, sleep disturbance, and social isolation. *OR* Odds ratio, *CI* Confidence interval

## Discussion

The present study examined the association between LBP and physical function among the elderly who were affected by the GEJE using 4-year longitudinal data. The study revealed that LBP was significantly associated with low physical function among the study participants, and the association became stronger as the duration or frequency of LBP increased. Further, preceding LBP was significantly associated with the onset of low physical function, and the association became stronger as the duration or frequency of preceding LBP increased.

Some cross-sectional studies showed an association between LBP and functional disability in the elderly [2, 12, 14, 34]. Leveille et al. reported that older women with severe LBP had more difficulty performing light housework and shopping than those with no or mild LBP [14]. Further, Rudy et al. showed that the elderly with chronic LBP had greater limitations in physical activities such as walking, getting up from the chair, and climbing stairs than those without chronic LBP [12]. The present study also showed that the elderly with LBP had a higher rate of low physical function than those without LBP, even after adjusting for potential confounding factors. Although several factors may be associated with functional decline [6, 21, 22], LBP is considered to be independently associated with functional disability in the elderly. Further, although reports assessing the dose-dependent association between LBP and functional disability are rare, Weiner et al. reported that LBP is associated with difficulty in functional tasks such as carrying or pulling items, gardening, and walking, which was stronger with increased intensity or frequency of LBP [34]. The present study also showed that the association between LBP and low physical function was stronger as the duration of LBP increased, which was significant in LBP duration of > 2 years. Long-lasting LBP is associated with functional disability in the elderly. Further, participants with more history of LBP had a higher rate of low physical function. LBP is associated with functional disability, and such association is observed to be stronger in a dose-dependent manner, which includes the intensity, frequency, and duration of LBP.

Some longitudinal studies have reported that musculoskeletal pain is associated with the onset of functional disability [35, 36]. On the other hand, the association between preceding functional disability and onset of musculoskeletal pain has also been reported [37]. Musculoskeletal pain and functional disability are considered to have an interactive relationship. When focusing on LBP, only a small number of longitudinal studies have assessed the association between preceding LBP and functional disability in the elderly [11, 26, 38]. A previous long-term longitudinal study showed that LBP was a predictor of functional disability 19 years later [38]. On the other hand, the 1-year longitudinal study showed that the association between preceding LBP and onset of functional disability was not significant. Reid et al. reported that restricting LBP lasting over 4 months was associated with decline in lower-extremity physical function 18 months later. The present study also showed that preceding LBP was associated with the onset of low physical function 2 years later. LBP is common but often self-limited and repeats over long periods [28, 39]. LBP is considered to affect the elderly’s physical function over time. Further, to the best of our knowledge, the present study is the first to report that the association between preceding LBP and the onset of low physical function was stronger as the duration or frequency of LBP increased. Musculoskeletal pain causes muscle weakness, reduced range of joint motion, and reflex inhibition of muscles, leading to gait instability [36, 40]. Such effects of pain on functional disability are considered to become stronger with prolonged or repetitive LBP. In an aged society, treating and preventing LBP are important to achieve a better quality of life and to subsequently prevent functional disability. Some exercise programs for preventing functional disability in the elderly have been attempted and shown to be effective [19, 20]. Additionally, exercise has also been reported to be useful in alleviating LBP [41]. A rehabilitation program aiming at not only preventing functional disability but also reducing LBP may be more effective in maintaining the elderly’s functional ability, which should be examined in future studies.

The present study has some limitations. The follow-up rates at the second and third time points were 76.6 and 84.9%, respectively. Information on non-responders was not obtained, which may have affected the results of this study. Second, this study assessed the association between preceding LBP and the onset of low physical function using longitudinal data. A reverse association is also possible and should be assessed in future studies. Finally, the present study used the data of people living in disaster-affected areas, and the generalizability of the results is not clear.

## Conclusion

LBP is significantly associated with low physical function in the elderly, and such association becomes stronger as the duration or frequency of LBP increases. Additionally, preceding LBP is significantly associated with the onset of low physical function in the elderly, and the effect becomes stronger as the duration or frequency of LBP increases.

## Supplementary Information

**Additional file 1.** .

## Data Availability

All relevant data are included in this article.

## Abbreviations

BMI: Body mass index
GEJE: Great East Japan Earthquake
KCL: Kihon Checklist
LBP: Low back pain
OR: Odds ratio
95% CI: 95% confidence interval
