# Correction: Association between low back pain and functional disability in the elderly people: a 4-year longitudinal study after the great East Japan earthquake

**DOI:** 10.1186/s12877-022-03715-y

**Published:** 2023-02-09

**Authors:** Yutaka Yabe, Yoshihiro Hagiwara, Yumi Sugawara, Ichiro Tsuji

**Affiliations:** 1 Department of Orthopedic Surgery, Tohoku University School of Medicine, 1-1 Seiryo-machi, Aoba-ku, Sendai, Miyagi 980-8574 Japan; 2 Department of Health Informatics and Public Health, Division of Epidemiology, Tohoku University Graduate School of Public Health, 2-1 Seiryo-machi, Aoba-ku, Sendai, Miyagi 980-8575 Japan

**Correction: BMC Geriatrics 22, 930 (2022)**

**https://doi.org/10.1186/s12877-022-03655-7**

After publication of this article [1], the authors reported that the symbol “3“ (superscript) should have been "≥", in Table 2 (twice) and Table 4 (once), respectively.

The original article [1] has been corrected.
