# Supplementary material for: Association between low back pain and functional disability in the elderly people: a 4-year longitudinal study after the great East Japan earthquake
Source: BMC Geriatr. 2022 Dec 2;22:930. doi: 10.1186/s12877-022-03655-7 (PMC9716857; doi:10.1186/s12877-022-03655-7)
Supplement: Supplementary file 1 — Additional file 1. . [file 12877_2022_3655_MOESM1_ESM.docx]

# 【9】We ask you for your health condition.

## （１）Have you had symptoms in the last few days?


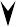


## ａ．yes ｂ．no

（２）（ the respondents who answer a. yes）How are the symptoms? Please circle all the ones that apply for your answer.

ａ．hand or foot pain ｂ．Irritated ｃ． head ache ｄ．dizziness ｅ. palpitation ｆ． Breathlessness ｇ．cough or sputum ｈ．wheezing ｉ． diarrhea

ｊ．constipation ｋ．anorexia ｌ．stomach ache

ｍ．pain or bleeding of hemorrhoid ｎ．toothache ｏ．Swelling or bleeding of gingiva

ｐ．difficulty in chewing ｑ．Itching (eruption・tinea etc.) ｒ．low back pain

ｓ．Incontinence of urine ｔ．swelling or languor of foot ｕ．difficulty in urinating・pain during urinating ｖ．Injury such as wound or burn

ｗ．menstrual irregularity・menstrual pain ｘ．fracture・sprain・dislocation

A．neck pain B．shoulder pain C．knee painｙ．other （ ）
